# Simultaneous Bilateral Intertrochanteric Fractures of the Femur: A Case Report and Review of the Literature

**DOI:** 10.7759/cureus.1236

**Published:** 2017-05-09

**Authors:** Raju Vaishya, Amit Kumar Agarwal, Ifeanyi C Nwagbara, Vipul Vijay

**Affiliations:** 1 Department of Orthopedics, Indraprastha Apollo Hospital, New Delhi; 2 Orthopaedics, Imo State University Teaching Hospital, Nigeria

**Keywords:** femur, complex, bilateral, inter-trochanteric, fracture, mechanism

## Abstract

Simultaneous bilateral intertrochanteric fractures are very rare, and only a few cases have been reported in the literature. They usually result from severe trauma. They are serious injuries with high morbidity and mortality rates. Successful management of these patients involves adequate resuscitation, treatment of associated injuries, early single-stage stable fixation, and good rehabilitation. We report the case of a 47-year-old male who presented with a simultaneous bilateral intertrochanteric fracture along with associated injuries following a car crash.

## Introduction

Fractures of the proximal femur are among the commonest encountered fractures, as this area of the body is subjected to the highest amount of load. Proximal femur is also commonly affected by pathological bone resorption. In the elderly population, these fractures occur following a trivial trauma; while in younger individuals, it occurs following a high-energy trauma [1]. These injuries are seldom bilateral but when they occur, the femoral neck is usually affected [2]. Simultaneous bilateral intertrochanteric fractures are rare, and only a few cases have been reported in the literature [3]. We present and discuss the management of a 47-year-old male with bilateral intertrochanteric fractures, with associated fractures of the left femoral shaft, left patella, right thumb, and ribs.

## Case presentation

**Figure 1:**
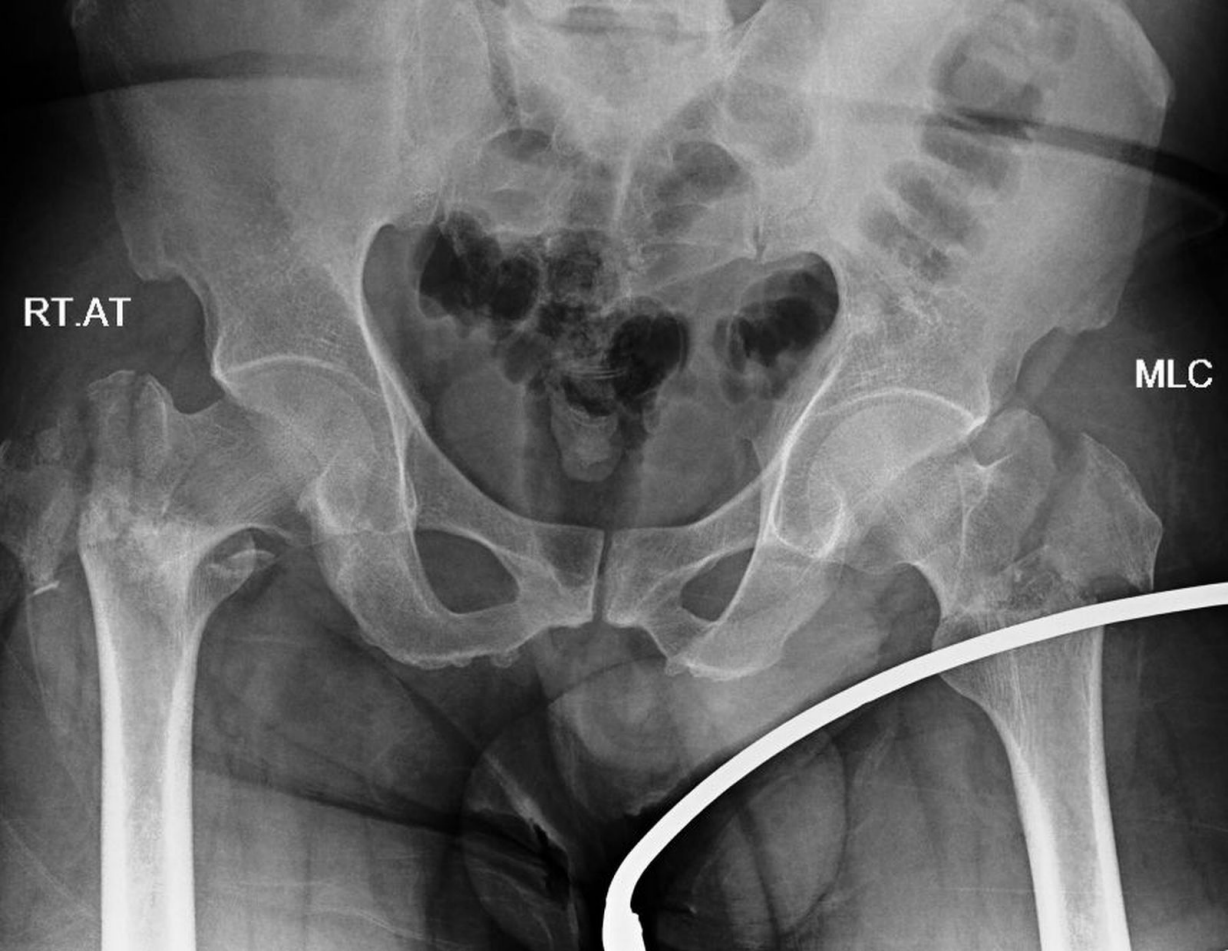
X-ray of the pelvis showing bilateral comminuted intertrochanteric fractures

**Figure 2:**
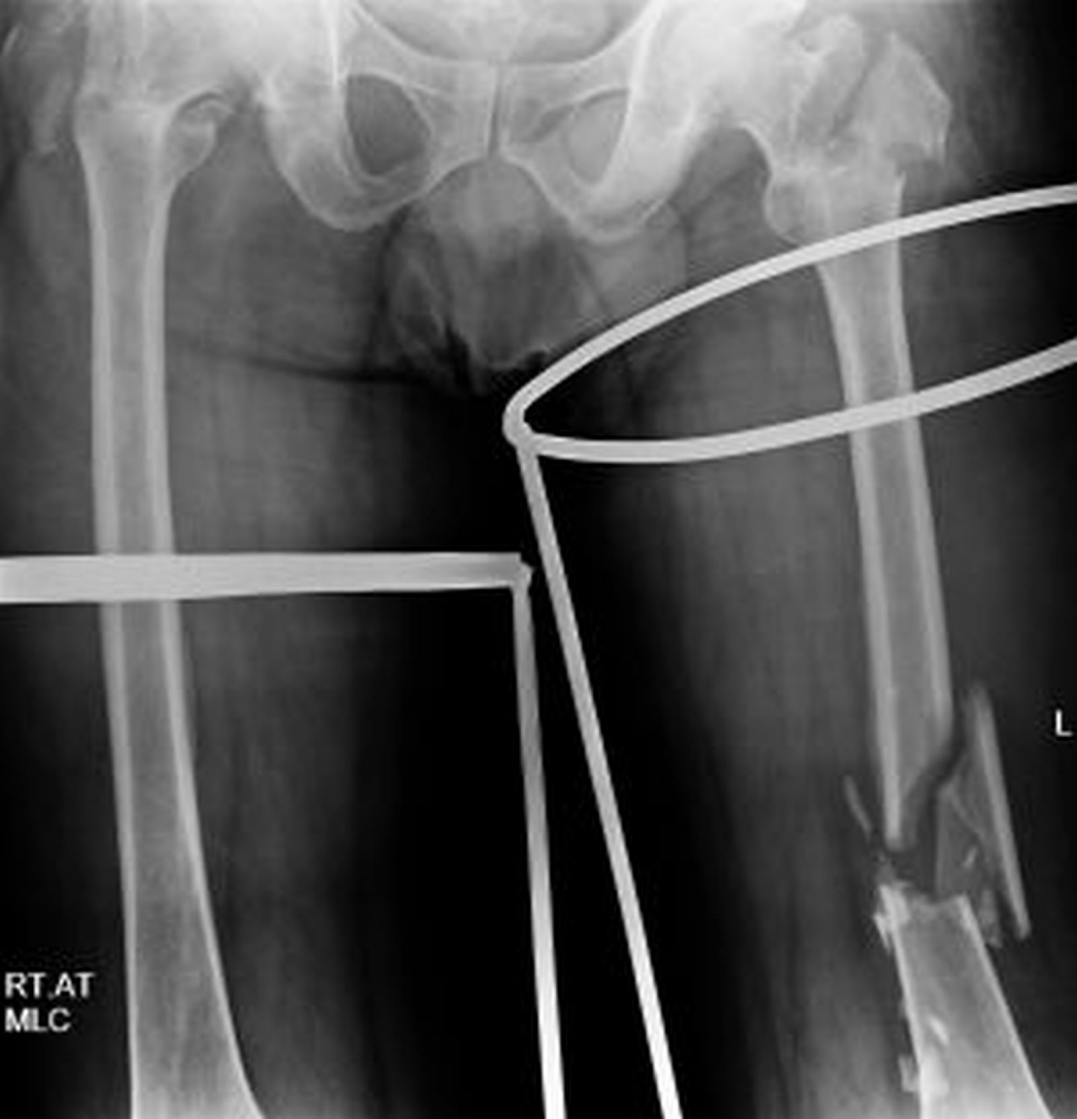
X-ray of the pelvis with both thighs showing comminuted left femoral shaft fracture

A 47-year-old male presented with pain in both the hips and an inability to bear weight on his legs. He sustained the injuries in a car crash three days back and took primary treatment at a local hospital. He had mild respiratory distress on presentation. The lower limbs were immobilised in the bilateral Thomas’s splints. The patient did not have any co-morbid conditions. The routine haematological and biochemical investigations were within normal limits. Oxygen saturation (SpO2) on room air was 90%, while it improved to around 97% with a 4-litre oxygen supply. The radiographs confirmed the presence of the following bone injuries - bilateral comminuted intertrochanteric fractures (Figure 1), comminuted left femoral shaft fracture (Figure 2), transverse left patella fracture, right 6th and 7th fractured ribs, and avulsion fracture of the distal phalanx of the right thumb.

**Figure 3:**
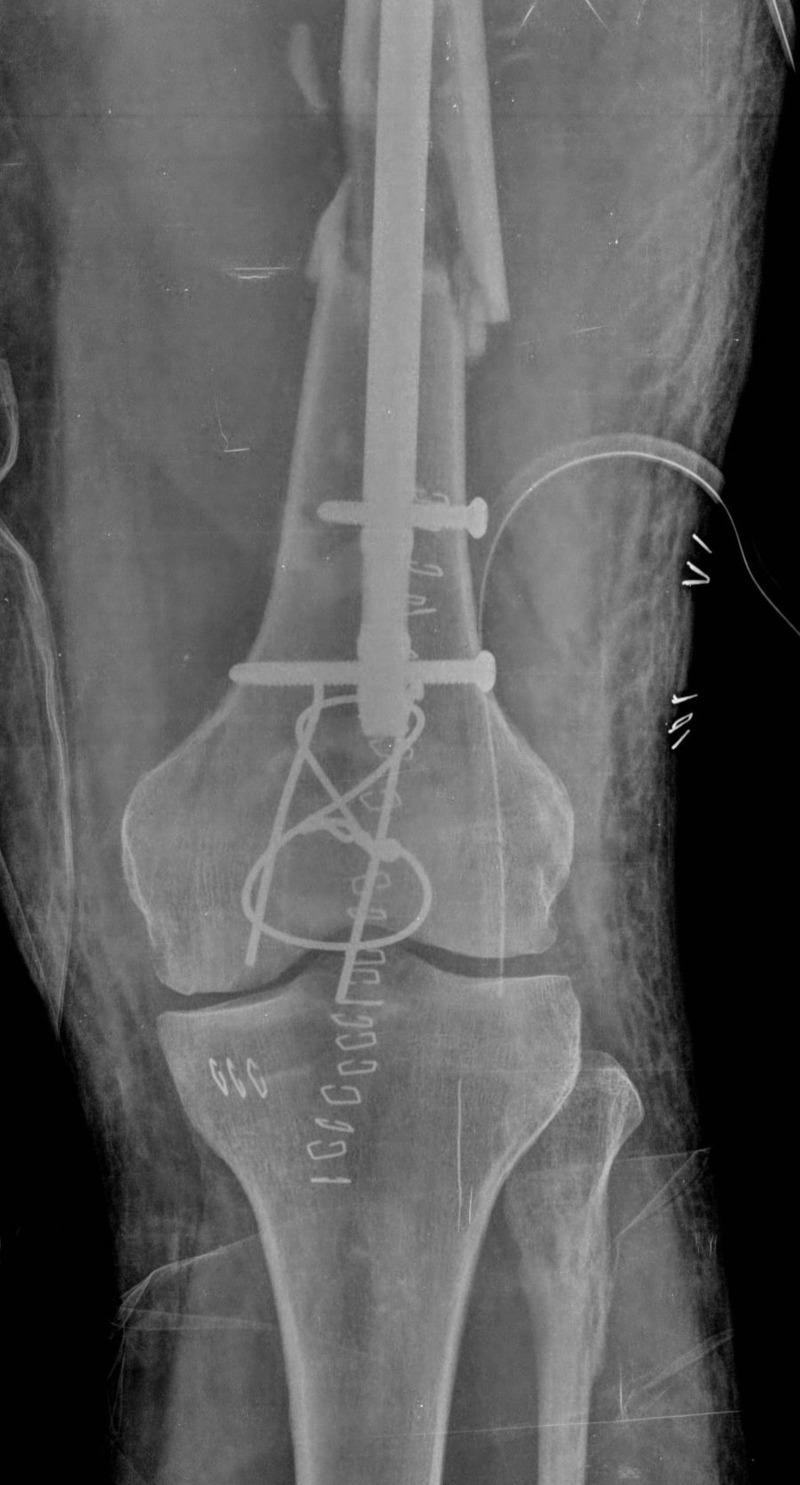
Immediate post-op antero-posterior x-ray of the patellar fracture with tension band wiring and femur interlocking

**Figure 4:**
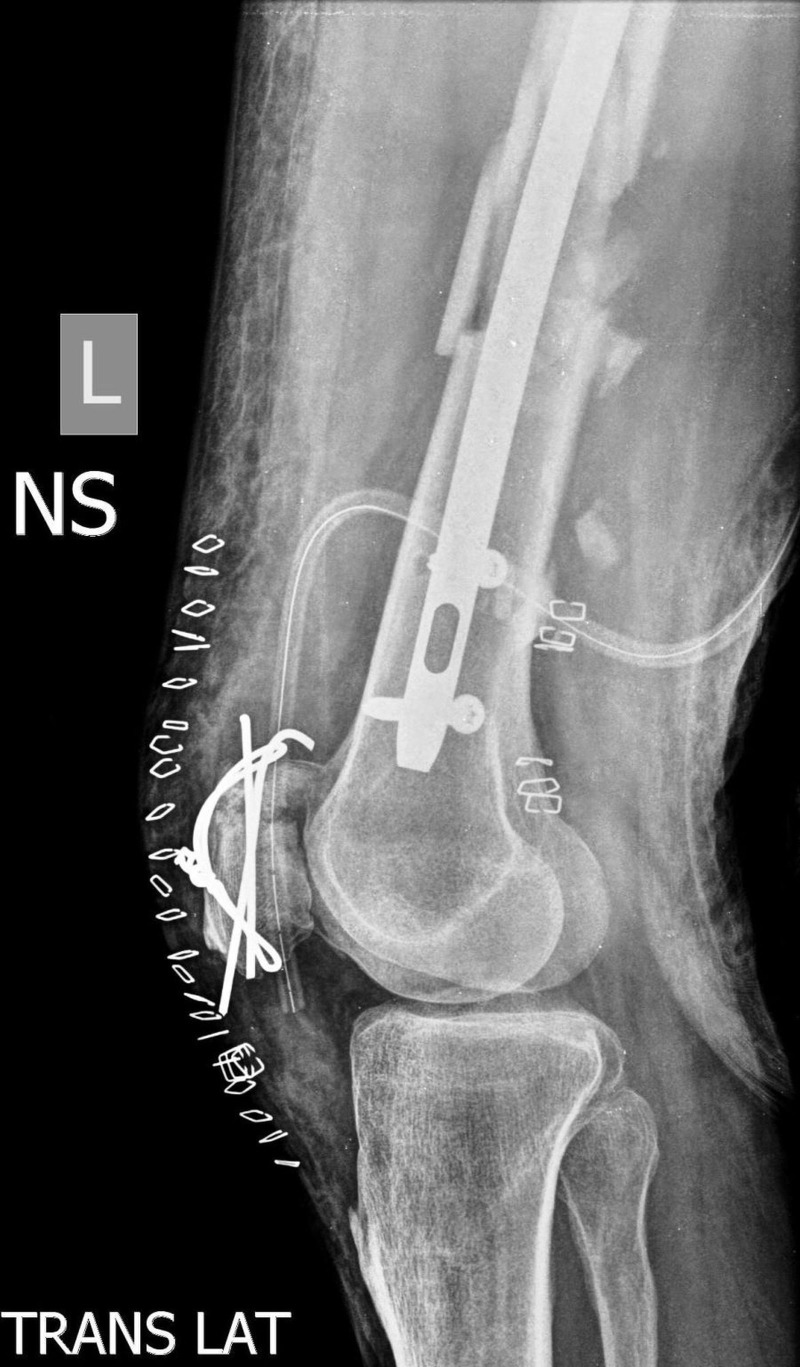
Immediate post-op lateral x-ray of the knee showing patella fracture fixed with tension band wiring

After optimizing the medical condition, his fractures were internally fixed under general anaesthesia. The fractures were fixed using a fracture table and an image intensifier. All the fractures were fixed in the same sitting. The left femoral fractures were managed with an intramedullary nail and interlocking screws (A2FN; SynthesTM, Switzerland), while the patella fracture was fixed with tension band wiring (Figures 3-4). The right intertrochanteric fracture was fixed with a five-hole reversed distal femoral locking plate because of severe comminution of the trochanteric region. The thumb fracture was internally fixed using a 1.3 mm suture anchor.

**Figure 5:**
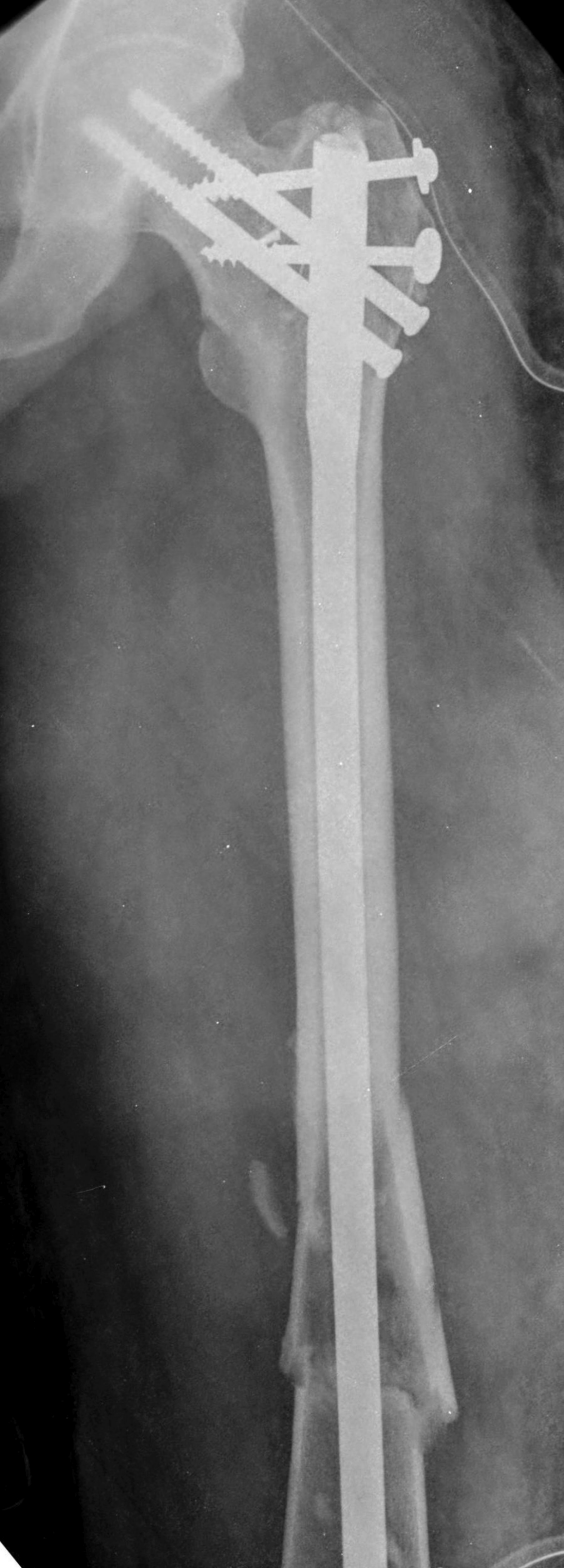
Six months post-op x-ray of the left femur showing union of the left trochanteric and femoral shaft fracture

**Figure 6:**
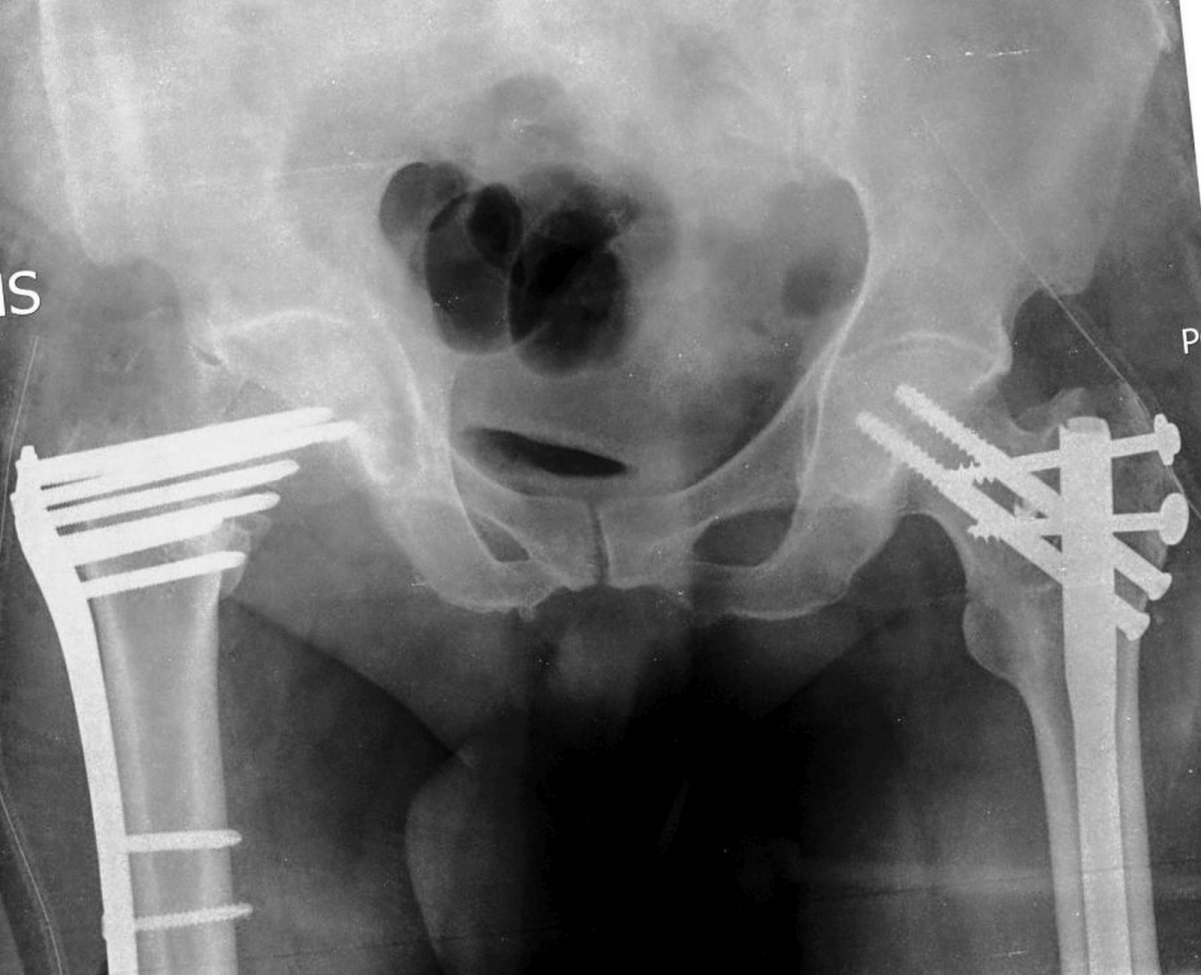
Six months post-op x-ray of the pelvis showing signs of healing of both the trochanteric fractures

The patient responded well following single-stage multiple fracture fixations. His postoperative period was uneventful. He was discharged on the 10th postoperative day in a stable condition. He was allowed mobilisation in bed immediately after surgery and on wheelchair one week after surgery. After two months, he was allowed weight bearing with the aid of a walking frame. At six months follow-up, the left femoral fractures had healed (Figures 5-6).

**Figure 7:**
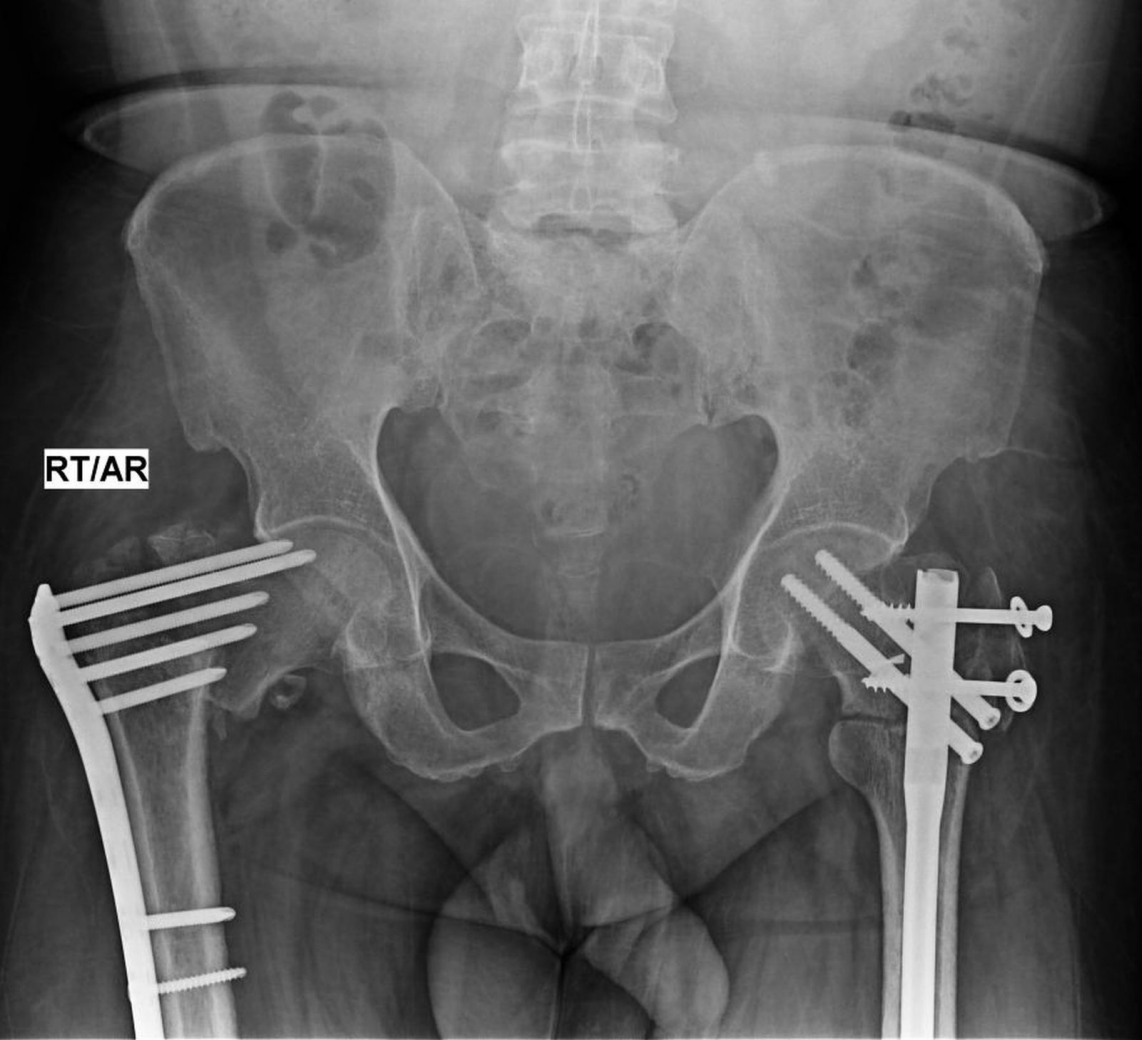
Nine months post-operative x-ray of the pelvis showing implant failure of the right hip

**Figure 8:**
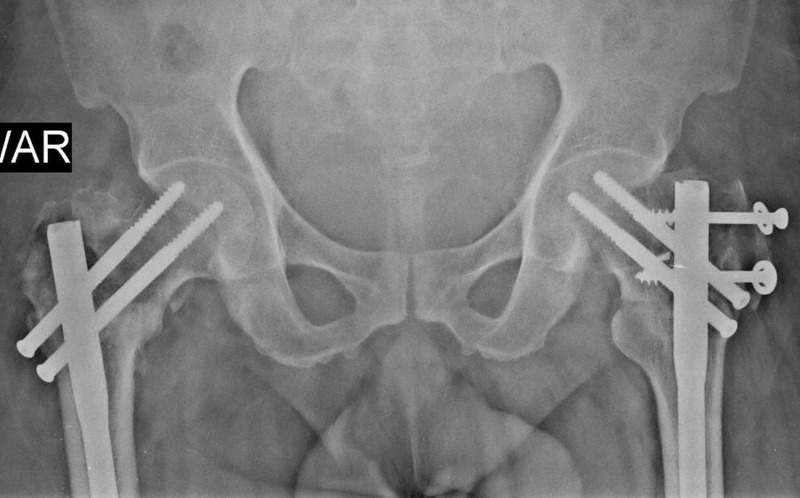
13 months post-operative x-ray of the pelvis showing union of both the fractures

Once the patient started to bear full weight on his right lower limb, the right femoral fracture fixation started to give way after nine months (Figure 7). The fixation of the right side was revised using an A2FN interlocking nail. The fracture healed in four months after the surgery (Figure 8). He had recovered about 80% of his movements of both the hip and knee.

## Discussion

Simultaneous bilateral intertrochanteric fractures are rare, and only a few cases have been reported in the literature. Its incidence in the general population is less than 0.3% [4]. This is in contrast to the unilateral variety that is much more commonly seen [5]. In young adults with good bone stock, a high energy impact is required to cause the bilateral intertrochanteric fractures. In contrast, only a minimal force is required to produce these fractures in an abnormal bone due to decreased elastic resistance as seen in osteoporosis, osteomalacia, and in primary or secondary neoplasia [6]. In a normal bone, the fracture is usually caused by a combination of violent rotational and compressive forces acting on both the lower limbs simultaneously. The injury mechanisms described in the literature include trapping of the lower limbs while the rest of the body continues to move at the time of impact, squeezing of the limbs in between vehicle parts, and run-over injuries [7].

Generalized convulsions as usually seen in epilepsy, metabolic disorders, and electrocution. Electroconvulsive therapy can also give rise to these fractures. During violent convulsions, the pelvi-trochanteric muscles simultaneously contract which then applies force to the femur and the pelvis irrespective of their relative positions [8].

Simultaneous bilateral intertrochanteric fractures are potentially life-threatening and result in fatal injuries. The affected patients are most likely to have other associated severe injuries because of the amount of force required to cause this type of fracture. Thus they should be managed based on the principles of treatment of multiple injuries. Careful assessment of the associated injuries and adequate resuscitation are important determinants of the survival of the patient. Attention should be given to the control of the hemorrhage and volume replacement with crystalloids and blood. Oxygen should be administered as required. Prophylaxis against deep venous thrombosis (DVT) and fat embolism should be instituted early in the management. While resuscitative measures are being conducted, the fractures should be splinted to make the patient as comfortable as possible. The timing of surgery is important. The patient should be fully resuscitated and stable before definitive treatment of the fractures is carried out. Surgery needs to be conducted as early as possible as this tends to significantly reduce the incidence of complications arising from the fracture and also shortens the period of hospitalisation [9].

A single-stage surgical procedure is advisable, and the aim is to achieve stable fixation of all the fractures to enhance nursing care and encourage early mobilisation of the patient. Both the surgeon and the anaesthetist should realise the task at hand as the procedure involves doing two or more major surgeries at the same sitting. The duration is likely to be prolonged, and arrangements should be made for an adequate amount of blood for transfusion. 

The selection of the fixation method depends on the fracture configuration and associated injuries. The available treatment options include the use of intramedullary nail, proximal femoral nail anti-rotation (PFNA), trochanteric femoral nail (TFN), dynamic hip screw (DHS), and an angle blade plate. A DHS with the plate is chosen in stable fractures. For unstable fractures and those with the loss of medial cortical support, PFNA, TFN, or angle blade plate is used [10]. An intramedullary nail is utilised when there is an associated femoral shaft fracture. In the index case, we used the intramedullary nail on the left side because of the associated femoral shaft fracture, and a reversed distal femoral locking plate on the right side due to severe comminution of the left greater trochanter.

The follow-up period in bilateral trochanteric fractures can be challenging for the patient. They have a longer hospital stay when compared to patients with unilateral fractures. One study puts the hospital stay at an average of 24 days as against 6.5 days for those with unilateral fractures. An early recovery is enhanced by utilising the right fixation method that achieves adequate stability to allow mobilisation as soon as possible. The patient is initially mobilised on a wheelchair but should be able to commence assisted weight-bearing as early as six weeks.

## Conclusions

Simultaneous bilateral intertrochanteric fractures are rare injuries. They are usually caused by high-energy trauma and are often associated with other fractures and injuries. These injuries are potentially life-threatening, and they are challenging to treat. Adequate resuscitation followed by single-stage stable fixation of multiple fractures is advisable. The implant that achieves the best possible stability should be used. 
